# Delinquent Mortgages, Neglected Swimming Pools, and West Nile Virus, California

**DOI:** 10.3201/eid1503.081489

**Published:** 2009-03

**Authors:** Richard A. Goodman, James W. Buehler

**Affiliations:** Centers for Disease Control and Prevention, Atlanta, Georgia, USA (R.A. Goodman); Emory University, Atlanta (J.W. Buehler)

**Keywords:** West Nile virus, mosquitoes, surveillance, mortgages, swimming pools, California, letter

## To the Editor

Reisen et al. illustrated the potential relationship between environmental developments, such as those that result from major economic events, and increased risks for infectious diseases (*1*). The Institute of Medicine landmark report in 1992 on emerging pathogens (*2*) and the initiative of the Centers for Disease Control and Prevention in 1994 (which helped give rise to the Emerging Infectious Diseases journal) note the role of environmental phenomena in spawning some infectious diseases.

The Reisen et al. report also reflects the utility and limitations of ecologic studies and the legitimacy of simultaneously concluding that study findings are “hypothesis generating” from a scientific perspective yet sufficiently plausible to prompt public health interventions from a practical perspective. The report demonstrates the value of synthesizing multiple streams of surveillance data, observations from field investigations, and contextual awareness of community events to generate hypotheses that bear exploration—in this case, the hypothesis that increases in mosquito habitats resulting from abandonment of swimming pools cause increases in the incidence of West Nile virus (WNV) cases. Scientifically, this question was not definitively answered because the study could not directly assess the putative link between disease and exposures to WNV-infected mosquitoes that had bred in abandoned swimming pools in California.

Beyond a priori knowledge that abandoned swimming pools provide opportunities for mosquito breeding (and other hazards, such as drowning), this report raises other critically important questions, such as what action should be taken in response to such findings and how should the findings be communicated to the public? Practically, questions about the link between abandoned swimming pools and illness were sufficiently answered so that public and environmental health officials could respond, as manifested by Kern County’s West Nile Virus Strategic Response Plan (*3*) and related strategies, including the Fight the Bite campaign, which calls for reporting of neglected swimming pools (*4*). Knowledge regarding the study’s limitations about causality should not detract from these efforts, as has been recognized by other sectors, such as the mortgage industry (*5*).
