# Supplementary material for: Delinquent Mortgages, Neglected Swimming Pools, and West Nile Virus, California
Source: Emerg Infect Dis. 2009 Mar;15(3):508–9. doi: 10.3201/eid1503.081489 (PMC2681132; doi:10.3201/eid1503.081489)
Supplement: Related texts

# Reply: In Response

**Authors:** William K. Reisen, Richard M. Takahashi, Brian D. Carroll, Rob Quiring

**Affiliations:** University of California, Davis, California, USA (W.K. Reisen, B.D. Carroll); Kern Mosquito and Vector Control District, Bakersfield, California, USA (R.M. Takahashi, R. Quiring)

We thank Goodman and Buehler for their comments about our recent article drawing attention to linkages among neglected swimming pools, vector mosquito production, and West Nile virus (WNV) transmission (*1*). We agree with their comment that our “study could not directly assess the putative link between disease and exposures to WNV-infected mosquitoes that had bred in abandoned swimming pools in California.” However, outbreaks of pathogens with complicated transmission cycles rarely afford isolation of any one factor for analysis. Notably, an update of events in Kern County during 2008 may support our previous conclusions. As in 2007, Kern County experienced continued drought conditions in 2008, and mosquito production was again confined largely to urban sources, including swimming pools. Avian abundance was low, and seroprevalence for WNV among selected bird species was similar to seroprevalence rates for 2007 (i.e., 7.8% of 192 house finches, 5.6% of 90 house sparrows, and 41.2% of 68 western scrub-jays were seropositive in 2007). However, early in the spring of 2008, unlike in 2007, the Kern Mosquito and Vector Control District (MVCD) commenced aerial surveys, reassigned rural field crews to treat swimming pools (and other urban water sources), and immediately conducted ground-based adulticiding when surveillance detected enzootic activity. This aggressive approach resulted in 2,182 swimming pools treated (a 255% increase over the number treated in 2007), reduced enzootic transmission (i.e., 5% of 192 dead birds, 17 serum samples from 90 sentinel chickens, and 1% of 598 mosquito pools positive for WNV), and almost no evidence of human infection (only 1 case reported in October). Intervention by the Kern MVCD, coupled with continuing severe drought conditions that eliminated natural and agricultural-related mosquito production sites, effectively eliminated WNV epidemic transmission that had persisted during the 4 previous years (*2*).

## References

[1] Goodman RA, Buehler JW. Delinquent mortgages, neglected swimming pools, and West Nile virus, California. Emerg Infect Dis. 2009;15:508. 10.3201/eid1503.08148919239785PMC2681132

[2] Reisen WK, Carroll BD, Takahashi RM, Fang Y, Garcia S, Martinez VM, Repeated West Nile virus epidemic transmission in Kern County, California, 2004–2007. J Med Entomol. In press.10.1603/033.046.0118PMC272946019198528
